# Solving Single Machine Total Weighted Tardiness Problem with Unequal Release Date Using Neurohybrid Particle Swarm Optimization Approach

**DOI:** 10.1155/2015/838925

**Published:** 2015-06-23

**Authors:** Tarik Cakar, Rasit Koker

**Affiliations:** ^1^Industrial Engineering Department, Engineering Faculty, Sakarya University, Esentepe Campus, 54187 Sakarya, Turkey; ^2^Electrical and Electronics Engineering Department, Faculty of Technology, Sakarya University, Esentepe Campus, 54187 Sakarya, Turkey

## Abstract

A particle swarm optimization algorithm (PSO) has been used to solve the single machine total weighted tardiness problem (SMTWT) with unequal release date. To find the best solutions three different solution approaches have been used. To prepare subhybrid solution system, genetic algorithms (GA) and simulated annealing (SA) have been used. In the subhybrid system (GA and SA), GA obtains a solution in any stage, that solution is taken by SA and used as an initial solution. When SA finds better solution than this solution, it stops working and gives this solution to GA again. After GA finishes working the obtained solution is given to PSO. PSO searches for better solution than this solution. Later it again sends the obtained solution to GA. Three different solution systems worked together. Neurohybrid system uses PSO as the main optimizer and SA and GA have been used as local search tools. For each stage, local optimizers are used to perform exploitation to the best particle. In addition to local search tools, neurodominance rule (NDR) has been used to improve performance of last solution of hybrid-PSO system. NDR checked sequential jobs according to total weighted tardiness factor. All system is named as neurohybrid-PSO solution system.

## 1. Introduction

Particle swarm optimization (PSO) is an evolutionary computation technique developed by Eberhart and Kennedy [1]. Its working principle is based on modelling the motion style of the birds. GA is a search technique based on population. The algorithm operates on the given population along the search procedure. There is no filtering procedure. Because of this feature, it differs from genetic algorithms. In this study, a hybrid system based on PSO has been proposed to solve single machine total weighted tardiness problem with unequal release date. Only one job can proceed in the single machine system that will be solved. It has a processing time, a due date, penalty of tardiness, and release date for each job. There is no preemption. The single machine total weighted tardiness problem with unequal release date can be seen in the manufacturing industry, chemical process industry, electronic and computer engineering, service systems, and many areas as well. One of the most studied scheduling problems is single machine total weighted tardiness (SMTWT). Computation method of SMTWT can be seen in Table 4. SMTWT with unequal release date problem is an NP-hard. Recently, some metaheuristics such as particle swarm optimization (PSO), genetic algorithm (GA), simulated annealing (SA), and ant colony optimization (ACO) have been applied to solve the single machine scheduling problems.

Generally, the exact solution of SMTWT problem can be done by using branch-and-bound algorithm (Kan et al. [2], Potts and Van Wassenhove [3], and Abdul-Razaq et al. [4]) and dynamic programming (Held and Karp [5] and Baker and Schrage [6, 7]). Another effective heuristic method is adjacent pairwise interchange (API) method to minimize mean tardiness. API was developed by Fry et al. [8].

Single machine total weighted tardiness problem with unequal release date is presented as follows: 1 |*rj*|Σ*wiTi*. A new dominance rule for 1 |*rj*|Σ*wiTi* problem can be used in reducing the number of alternatives in any exact approach by Akturk and Ozdemir [9]. A neurodominance rule has been used for single machine tardiness problem with unequal release dates by Cakar [10]. Mahnam and Moslehi [11] studied on the problem of the minimization of the sum of maximum earliness and tardiness on a single machine with unequal release times in their paper. It has been proven that this problem is NP-hard in the strong sensation and a branch-and-bound algorithm has been developed as an exact method. Eren [12] considered single machine scheduling problem with unequal release dates and a learning effect in his paper. The problem of scheduling *n* jobs with release dates, due dates, weights, and equal process times on a single machine has been studied by Van den Akker et al. [13]. The target is the minimization of total weighted tardiness. Kooli and Serairi [14] solved the SMTWT with unequal release date using mixed integer programming approach. Yin et al. [15] used several dominance properties and branch-and-bound algorithm with honey bees optimization algorithm (MBO) to solve the SMTWT with unequal release date. Wu et al. [16] applied simulated annealing approach to solve the SMTWT with unequal release date.

Matsuo et al. [17] used simulated annealing algorithm for single machine total weighted tardiness (SMTWT) problem. Crauwels et al. [18] presented a comparative study of a few heuristic methods such as TS (Tabu search), GA, and SA for SMTWT problem. The best one among these heuristics was TS. Den Besten et al. [19] and Merckle and Middendorf [20] used ant colony optimization (ACO) for SMTWT problem. Laguna et al. [21] presented a paper about the discussion of the use of three local search strategies within a Tabu search method for the approximate solution of a single machine scheduling problem. Cheng et al. [22] proposed a hybrid algorithm to minimize total tardiness based on ACO metaheuristic.

Tasgetiren et al. [23] used PSO to solve SMTWT problem; furthermore, ACO and ILS (iterative local search) have been compared in his study. Panneerselvam [24] proposed a simple heuristic to solve single machine scheduling problem. Sen et al. [25] published a detailed survey paper about SMTWT. Additionally, a review study about SMTWT has been done by Koulamas [26]. Yang et al. [27] proposed a combined approach with PSO and SA to improve performance of PSO.

PSO, GA, and SA are search algorithms based on different search topologies. Since each method has different search mechanisms, obtained best solutions and the steps to reach the best solution may differ. But if these algorithms are used as a hybrid system in other words as a combined system, the quality of the obtained best solution and the process time are improved. Therefore, GA and SA are used together with PSO based algorithm. Designed search system that consists of GA and SA is named as subhybrid solution system and works interactively to search for the best solution. Here, SA supports GA. SA gets the best solution found by GA as initial solution and when it finds better solution than this solution, it transfers this better solution to GA. When SA supported GA and subhybrid solution system, finds the best solution, and stops working, then it sends this obtained best solution to PSO algorithm. PSO gets this best solution into its population and tries to find better solution. The system including subhybrid solution system and PSO is named as hybrid-PSO system. Then, when PSO finishes working, it sends the obtained solution again to GA and the solution loop will keep going like this. This continuous working system also prevents PSO to stop in any local minima. Sometimes all solutions in the swarm may be the same and to escape from this unwanted situation different algorithms are used to support PSO. Here in the proposed solution system PSO has no chance to stop in any local minima since subhybrid solution system based on combination of GA and SA is used. When the final solution is obtained by hybrid solution system, neurodominance rule (NDR) is applied to this solution. NDR checks the obtained final solution if there is any one violating other one's order and if there is, it warns. The criterion of NDR about changing sequentially coming jobs is TWT criterion. This overall solution system is named as neurohybrid-PSO system. In this study, the performances of PSO and neurohybrid-PSO systems have been compared. It is observed that neurohybrid-PSO has better performance.

This paper is organized as follows.  Section 2 explained the computational structure of PSO. In Section 3, solution steps and used parameters of PSO are discussed.  Section 4 shows the explanation of how SPV rule works.  Section 5 discussed genetic algorithms.  Section 6 discussed simulated annealing algorithm.  Section 7 shows the working structure of neurodominance rule presented. In Section 8, neurohybrid-PSO solution system is explained with working mechanisms. In Section 9, experimental design, computational results, and analysis about neurohybrid-PSO solution system are reported.

## 2. Computational Structure of Particle Swarm Optimization

Particle swarm optimization (PSO) is a population based evolutionary algorithm found by Russell Eberhart and James Kennedy in 1995. This algorithm has been modelled based on the actions of the bird and the fish swarms when they are looking for food and how they are escaping from any dangerous case. Pseudo code of PSO can be seen in Algorithm 1. Since PSO finds solution faster, requires less parameters, and lacks possibility of stopping in local minima, it has superiority on other algorithms.

PSO consists of the elements named as particle, where each particle generates different solution alternatives to the related problem. These particles community is named as swarm. All particles in the swarm begin to search for process by getting random values in the solution space. Each particle has two vectorial components that are position (*P*) and velocity (*v*) vector. The position vector keeps the position information of the particle and the velocity vector keeps amount of the displacement and direction of the particle. In PSO, which is an iterative algorithm, the velocity components and thereby position component are updated in each iteration. The new velocity value of a particle is calculated by using the experience obtained in the previous iterations, the general experience of the swarm and randomness:

(1) vzki+1=μvzki+c1R1Pbestzki−Xzki+c2R2Gbestki−Xzki,

where *i*: is the number of iterations, *c*_1_ is a self-learning factor, *c*_2_ is a social learning factor, *R*_1_, *R*_2_ are randomly generated numbers between [0-1], *μ* is the inertia weight, *P*_best_ is the best position value found by particle in the latest iteration and named as local the best value, and *G*_best_ is the best position value in the swarm found until that time and named as global the best value. The new velocity value of the particle is calculated by using the previous velocity value and the local best value and global best value.

The new position vector is computed by adding the new position vector value to the old position vector as shown in

(2) $$\begin{array}{c} X_{zk}\left(\;i+1\;\right)=X_{zk}\left(\;i\;\right)+v_{zk}\left(\;i+1\;\right). \end{array}$$

The position values of particles are taken and the quality of proposed solutions is determined by using fitness function. The fitness function is an evaluation function, which gets the position values of particles as input parameters and generates numerical values. In the minimizations problems the particles having smaller fitness values are preferred to the particles having greater fitness values; on the other hand, in the maximization problems, the particles having greater fitness values are preferred to the particles having smaller fitness values, reversely.

As a result, each particle in PSO is started to search for random position and velocity values. In each iteration, the velocity and position values are updated and a fitness value is generated by using fitness function. Additionally, in each iteration the best local value of the particle and the best global value of the swarm are updated. After a certain number of iterations the best value of the swarm will be the solution presented by PSO algorithm for the given problem [28].

## 3. Solution Steps and Used Parameters of Particle Swarm Optimization Algorithms

**Step 1 .**

Assigning initialization values, one has to do the following.

- Set *i* = 0 as iteration counter starting value.
- Generate particles as randomly.

The continuous values belonging positions are randomly established. The following equation is used for the uniformly construction of the initial continuous position values belonging to the particle:

(3) $$\begin{array}{c} X_{zk}^{0}=X_{\text{MIN}}+\left(\;X_{\text{MAX}}-X_{\text{MIN}}\;\right)*R_{1}. \end{array}$$

In this equation, *X*_MIN_ = −5.0, *X*_MAX_ = 5.0, and *R*_1_ is a uniform random number between 0 and 1. *R*_1_ = *E*(0,1):

(4) $$\begin{array}{c} X_{ZK}=\left[\;X_{\text{MIN}},X_{\text{MAX}}\;\right]=\left[\;-5.0,5.0\;\right]. \end{array}$$

Population size has been taken as 30.

(C) Initial velocities are generated by a similar formula as follows:
  (5) $$\begin{array}{c} V_{zk}^{0}=V_{\text{MIN}}+\left(\;V_{\text{MAX}}-V_{\text{MIN}}\;\right)*R_{2}. \end{array}$$

Continuous velocity values are restricted to some range:

(6) $$\begin{array}{c} V_{ZK}=\left[\;V_{\text{MIN}},V_{\text{MAX}}\;\right]=\left[\;-5.0,5.0\;\right]. \end{array}$$

*R*_2_ is a uniform random number between 0 and 1: *R*_2_ = *E*(0,1).

(D) Apply the smallest position value (SPV) to find a sequence performing personal best.
(E) Evaluate each particle in the swarm using fitness function. Compute the personal best (*P*_best_).
(F) Obtain the best fitness value (*G*_best_) comparing all of the personal best.

**Step 2 .**

Running the counter, one has

(7) $$\begin{array}{c} i=i+1. \end{array}$$

**Step 3 .**

Updating inertia weight, *α* is a decreasing factor:

(8) μi+1μi∗αµµMIN,µMAX=4.0,9.0Decrement  factor  α0.975.

**Step 4 .**

Updating velocity,

(9) vzki+1=μvzki+c1R1Pbestzki−Xzki+c2R2Gbestki−Xzki.

Social and cognitive parameters have been taken as *c*_1_ = *c*_2_ = 2, and *R*_1_ and *R*_2_ can be described as uniform random numbers between (0, 1).

**Step 5 .**

Updating position,

(10) $$\begin{array}{c} X_{zk}\left(\;i+1\;\right)=X_{zk}\left(\;i\;\right)+v_{zk}\left(\;i+1\;\right). \end{array}$$

**Step 6 .**

Find the sequence applying SPV rule.

**Step 7 .**

Compute the *P*_best_ using new sequences, compare previous personal best and current personal best, and select the successful particle.

**Step 8 .**

Update the global best (*G*_best_).

**Step 9 .**

Stopping criterion, if program reaches the maximum number of iterations, then stop.

## 4. The Application of the SPV Rule and Demonstration of a Particle

The solution space to be searched in SPO can be shown as a matrix. Each row of this matrix represents a particle, and it represents a job order for SMTWT problem with unequal release date. The total weighted tardiness of each job order gives personal best, and the best of them will give the global best:

(11) $$\begin{array}{c} X=\left[\begin{array}{cccc} x_{11} & x_{12} & \cdots & x_{1n}\\ x_{21} & x_{22} & \cdots & x_{2n}\\ \vdots & & & \vdots \\ \vdots & & & \vdots \\ \vdots & & & \vdots \\ x_{n1} & x_{n2} & \cdots & x_{nn} \end{array}\right]. \end{array}$$

A job order will be found according to *X* value by using SPV rule, and according to this job order TWT value will be calculated. Computation method of complete time can be seen in Figure 2.

Here, to find the job schedule the ordering is done starting from the smallest *X* value to the biggest *X* value and by this way *S*_*ij*_ job schedule is found. As it is seen from Table 1, the job schedule is as 4-6-5-8-1-7-2-3.

## 5. Genetic Algorithms

The genetic algorithms are search and optimization methods based on natural selection principles. The principles of the genetic algorithms have been firstly presented by John Holland. After the presentation of these fundamental principles of the genetic algorithms, many scientific studies have been published. The genetic algorithms, which are different from traditional optimization methods, do not use parameter set, but they use their coded forms. The genetic algorithms working based on probabilistic rules need only target function. They do not search for all of the solution space, but they only search for a certain part of the solution space. Thus, they implement an efficient search and reach the solution in a shorter time. Another important superiority of the GA is to examine the population composed of solutions simultaneously and not to stop in local solutions by this way.

The genetic algorithms do not deal with the problem, but they deal with their codes. The code modelling is done based on the structure of the problem. The initial population is formed. The operations are done based on determined crossover and mutation rates, and in each population the ranking is done based on the best fitness value. The algorithm is ended when the defined population number is reached, or the determined fitness value is obtained.

The application of GA for the solution of SMTWT problem with unequal release date can be described in the following form. Each chromosome represents one job order. The fitness function is TWT. Linear Order Crossover (LOX) method has been used as a crossover method. Working principle of LOX method can be seen in Cakar [29] and Cakar [10]. The determined rates and values regarding solution have been given below:

 crossover rate: 100%,
 mutation rate: 4%,
 number of the population: 250,
 population size: 100.

## 6. Simulated Annealing

Simulated annealing (SA) is a heuristic algorithm too much successfully applied to the combinatorial optimization problems. SA algorithm is a technic, which gets model and reference the annealing process of the melt metals during the cooling. The target function of the order of the produced solutions by this method will show a general decrement. However, due to the structure of the algorithm in some cases some solutions with higher target function values are also accepted. By this way the algorithm does not stop because of a local minimum solution and it will keep going the search or a better solution or for a better local minima. The SA algorithm is a useful heuristic search algorithm, which has given the solutions near the best solutions for especially combinatorial optimization problems.

**Figure 1:**
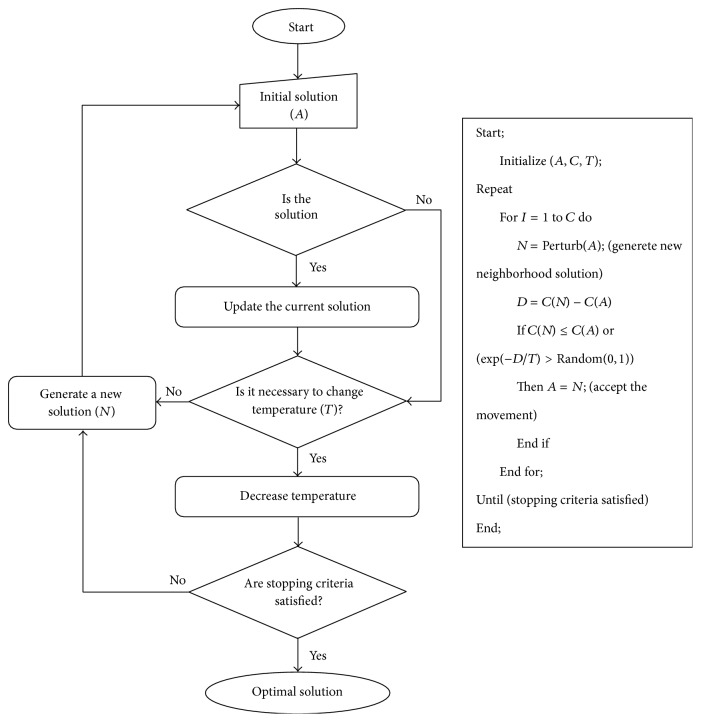
Flowchart and pseudocode of the simulating algorithm.

**Figure 2:**
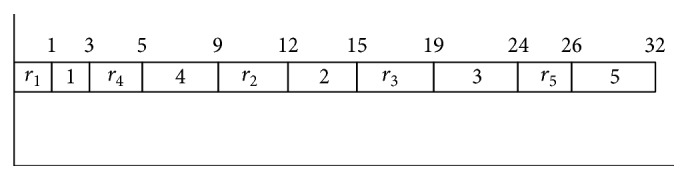
Gantt chart of the last job sequence.

In Figure 1 the flowchart and pseudocode of SA algorithm have been given [30, 31]. As seen in the figure, the SA is starting with an initial solution (*A*), initial temperature (*T*), and an iteration number (*C*). The role of the temperature is to control the possibility of the acceptance of the disturbing solution. On the other hand, the reason of the usage of the iteration number is to decide the number of repetitions until a solution is found on a stable state under the temperature [32, 33]. The temperature may get the following implicit flexibility index meaning. At the beginning of the searches, in other words, at high temperature situation, some flexibility may be moved to a worse solution cases; however, less of this flexibility is existing in the searches done later, which means at lower temperature. Based on these *T*, *C* through a heuristic perturbation on the existing solutions, a new neighborhood solution (*N*) is generated. In case of improvement on the change of an objective function, the neighborhood solution (*N*) will be a good solution. Even if the change of an objective function is not improved, the neighborhood solution will be a new solution with a suitable probability based on *e* ^−*D*/*T*^. This situation removes the possibility of finding a global optimum solution out of a local optimum. In case of no change after certain iterations, the algorithm is stopped. If there is still improvement on the new solution, the algorithm continues with a new temperature value.

To generate new solution two different operators have been used: swap and inverse operator. Swap operator is the same as mutation process of GA. During the inverse operator, a sequential job group is randomly chosen and then reversely ordered. Thus, a new solution alternative is obtained:

(12) Solution123456789New  Solution126543789.

## 7. Neurodominance Rule

Neurodominance rule is a system obtained based on training of a backpropagation neural network (BPANN) by using the data prepared with the implementation of API method. It is an intelligent system, which decides the priority of sequential two jobs based on TWT criteria. If any sequence violates the neurodominance rule, then violating jobs are switched according to the total weighted tardiness criterion.

The starting time of job *i*, the processing time of job *i*, due date of job *i*, the weight of job *i*, the processing time of job *j*, the due date of job *j*, the weight of job *j*, release date of job *i*, and release date of job *j* were given as inputs to the BPANN. “0” and “1” values were used to determine the precedence of the jobs. If output value of the BPANN is “0,” then *i* should precede *j*. If output value of the BPANN is “1,” then *j* should precede *i* [10].

Working mechanism of neurodominance rule has been explained with an example; see Tables 2 and 3.

## 8. Neurohybrid System Based on PSO

**Figure 3:**
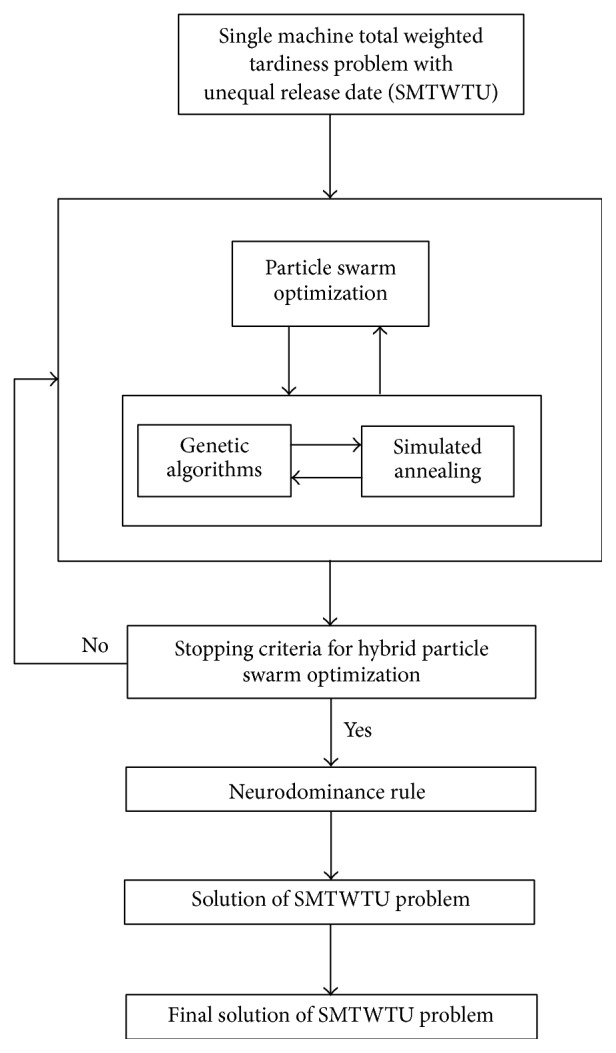
The structure of the proposed neurohybrid solution system based on PSO.

**Figure 4:**
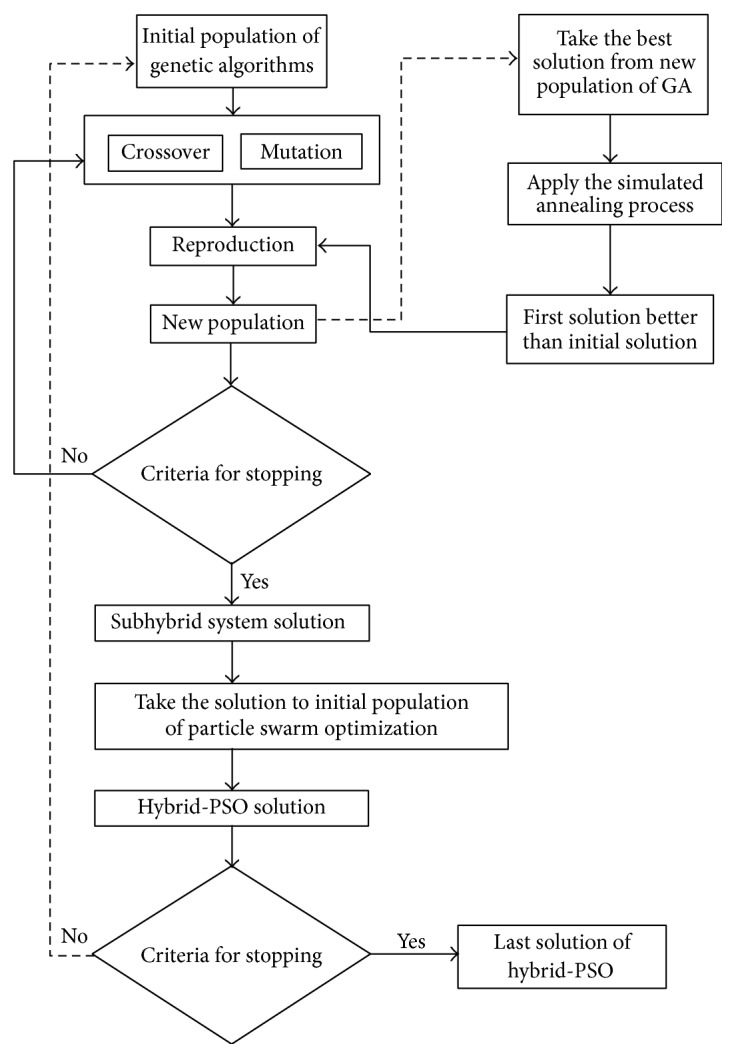
Working mechanism of hybrid-PSO system.

**Figure 5:**
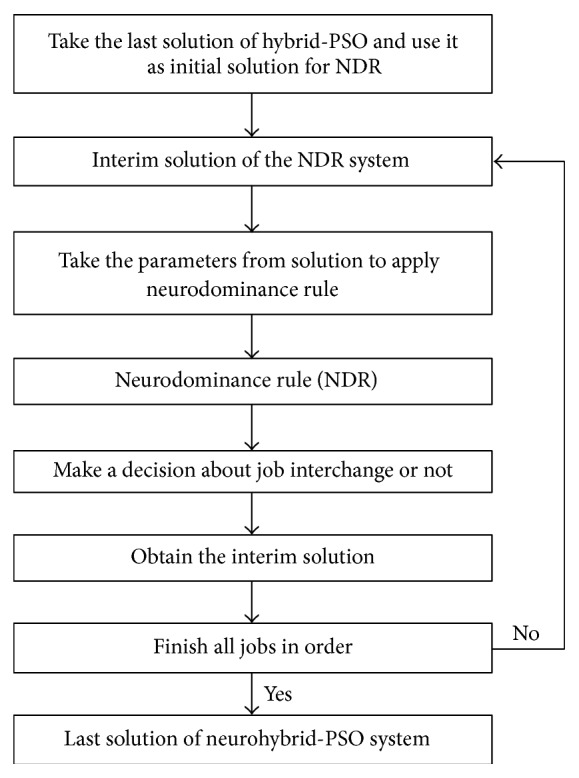
How neurodominance rule works as a part of neurohybrid-PSO solution system.

In this system PSO system works primarily and later it tries to improve the obtained solution by using GA and SA together as a hybrid system to find better solution. GA and SA work interactively. SA gets the best solution found by GA, and it improves this solution and sends the obtained better one to GA again. When working of GA finishes, obtaining the best solution by subhybrid solution is transferred to the initial population of PSO algorithm to search for better solution. The best solution found by PSO (hybrid-PSO solution) is sent to GA again, and GA and SA work interactively to search for a better solution. This loop stops if the obtained solution is fitting with predefined stopping criterion. As a result of working of these three search algorithms interactively, since each algorithm has different search mechanisms, faster and better solutions may be found. Then, neurodominance rule (NDR) is applied to obtained final solution and based on total weighted tardiness criterion, the violating orders are corrected, and the solution gets more excellent. The overall system is named as neurohybrid-PSO. The general working principle of the proposed solution system has been shown in Figure 3; on the other hand, detailed working system has been presented in Figures 4 and 5.

## 9. Experimental Design and Solutions

In this problem, the success of the PSO based neurohybrid system (NHPSO) has been repeated 100 times by using randomly generated 8100-sample set. Upper and lower bounding schemes have been used as performance measurement criteria. Processing times intervals, weights intervals, and the number of jobs have been shown in Table 5. And both of them are integers. The proportional range belonging due dates (RDD) and average tardiness factor (TFF) were selected from the following set: {0.1, 0.3, 0.5, 0.7, 0.9}. *d*_*i*_, an integer due date from the distribution [*P*(1 − TF − RDD/2), *P*(1 − TF + RDD/2)] was generated for each job *i*; here, *P* represents total processing time, ∑_*i*=1_^*n*^ *p*_*i*_. Release dates are produced based on a uniform distribution between 0 and *β*∑*p*_*j*_.

The COVERT, ATC, WSPT, WDD, WPD, EDD, LPT, SPT, and CR priority rules are primarily applied to the randomly generated problems. COVERT and ATC are dynamic priority rules and the others are static rules. The formula and working principle are as given below. The initial population of PSO has been constituted using the obtained solutions by using implementation of the priority rules mentioned above:

(1) COVERT
  (13) $$\begin{array}{c} \max\left[\;\frac{w_{i}}{p_{i}}\max\left(\;0,1-\frac{\max\left(0,d_{i}-t-p_{i}\right)}{kp_{i}}\;\right)\;\right]. \end{array}$$
(2) ATC
  (14) $$\begin{array}{c} \max\left[\;\frac{w_{i}}{p_{i}}\exp\left(\;-\frac{\max\left(0,d_{i}-t-p_{i}\right)}{k\bar{p}}\;\right)\;\right]. \end{array}$$
(3) EDD
  (15) $$\begin{array}{c} \min\left(\;d_{i}\;\right). \end{array}$$
(4) SPT
  (16) $$\begin{array}{c} \min\left(\;p_{i}\;\right). \end{array}$$
(5) LPT
  (17) $$\begin{array}{c} \max\left(\;p_{i}\;\right). \end{array}$$
(6) CR
  (18) $$\begin{array}{c} \min\left(\;\frac{d_{i}}{p_{i}}\;\right). \end{array}$$
(7) WSPT
  (19) $$\begin{array}{c} \max\left(\;\frac{w_{i}}{p_{i}}\;\right). \end{array}$$
(8) WDD
  (20) $$\begin{array}{c} \max\left(\;\frac{w_{i}}{d_{i}}\;\right). \end{array}$$
(9) WPD
  (21) $$\begin{array}{c} \max\left(\;\frac{w_{i}}{p_{i}d_{i}}\;\right). \end{array}$$
(10) FCFS
  (22) $$\begin{array}{c} \text{First Come First Served.} \end{array}$$

In Table 6, the average of the best values of PSO in the initial population and then the obtained best solutions by the implementation of PSO have been compared, and the amount of the improvement has been reported. Furthermore, NHPSO has been applied to the same problems and the amount of improvements comparing to the initial solutions have also been given. As it is evidently seen in Table 6, hybrid solution system has shown better improvement. Additionally, the contribution of the used NDR to the hybrid system is also demonstrated in Table 6. Also, comparison of GA and SA can be seen in Tables 7 and 8.

**Figure 6:**
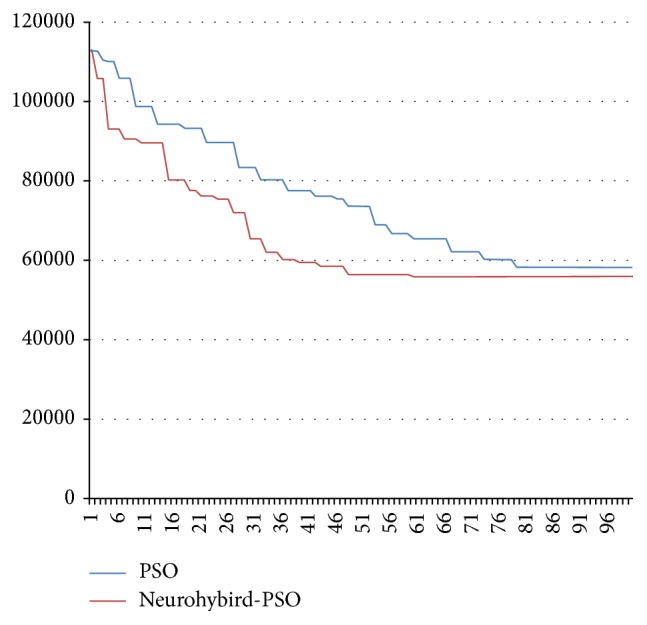
Comparison of PSO and NHPSO results according to total weighted tardiness factor for an example problem.

**Figure 7:**
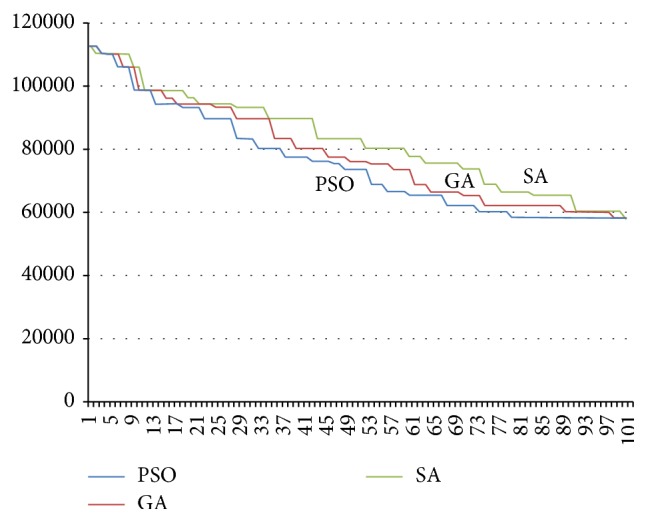
Comparison of PSO, GA, and SA for a SMTWT problem.

The solution values given by PSO and NHPSO for 100 generations have been presented in the graphical representation on Figure 6. It is evident that the proposed NHPSO is working better, reaching the solution quicker and giving better solution in a certain generation. These features are given to the NHPSO by interactive working SA, GA, and NDR applying these to the final solution. Comparison of PSO, GA, and SA can be seen in Figure 7.

The linear lower bound has been originally obtained by Potts and Van Wassenhove [34] based on using the Lagrangian relaxation approach with subproblems that are total weighted completion time problems. An additional derivation of it has been presented by Abdul-Razaq et al. [4] based on the reduction of the total weighted tardiness criterion to a linear function, that is, total weighted completion time problem. The parameters can be described as given in the following form for the job *I*: *I* = 1 to *n*, *w*_*i*_ ≥ *v*_*i*_ ≥ 0 and *C*_*i*_ is the completion time of job *I*, and the author has

(23) wiTiwimax⁡Ci−di,0≥vimax⁡Ci−di,0≥viCi−di.

Assume that *v* = (*v*_1_, …, *v*_*n*_) is a vector of linear weights, that is, weights belonging to the linear function *C*_*i*_ − *d*_*i*_, chosen so that 0 ≤ *v*_*i*_ ≤ *w*_*i*_. If so a lower bound can be written by using given linear function below:

(24) $$\begin{array}{c} LB_{LIN}\left(\;v\;\right)=\overset{n}{\underset{i=1}{\sum }}v_{i}\left(\;C_{i}-d_{i}\;\right)\leq \overset{n}{\underset{i=1}{\sum }}w_{i}max\left\{\;C_{i}-d_{i},0\;\right\}. \end{array}$$

This situation demonstrates that the solution of the total weighted completion time problem takes a lower bound on the total weighted tardiness problem. The lower bounding scheme has also been used by Akturk and Yildirim [35] and Cakar [10] in their studies.

In Tables 9 [Table tab10]–11, it is clearly seen that SA, GA, PSO, and NHPSO are improving the linear lower bound for the given different number of jobs. Proposed method NHPSO is doing improvement better than PSO, GA, and SA. Furthermore, NDR is also contributing to the improvement of lower bound here.

The number of better, equal, or worse lower bounds obtained for the given examples has been presented in Tables 12 [Table tab13]–14. The amount of improvement is noteworthy at %99.5 confidence level.

## 10. Conclusion

In this study, we proposed a neurohybrid-PSO system to solve total weighted tardiness problem with unequal release date. It is known that hybrid intelligent systems work better than the others. In the proposed hybrid system, GA and SA work interactively with each other to support PSO and increase the performance of the PSO algorithm. It has been shown in the paper that the proposed neurohybrid-PSO works better than PSO. NDR method has been applied to the solution obtained from hybrid-PSO which has been working interactively with GA and SA, to improve the solution more. Computational results showed that NDR improves the hybrid-PSO system's performance. It can be seen that neurohybrid-PSO solution system can improve the upper and lower bounding schemes. In the future, the proposed solution system may be applied to single machine total weighted tardiness problem with double due date.

## Figures and Tables

**Algorithm 1:**
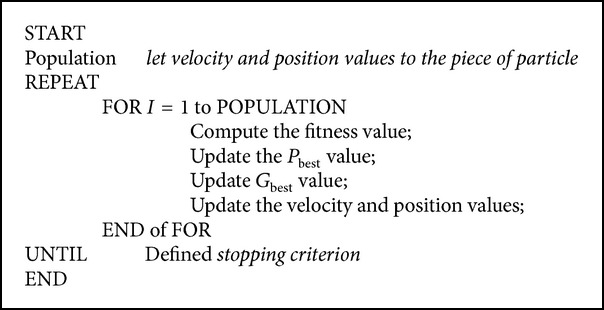
PSO algorithm.

**Table 1:** Solution analysis of a particle using NPV rule.

*J*	1	2	3	4	5	6	7	8

*X*_*ij*_	1.75	2.86	3.12	−0.97	1.12	−0.68	2.22	1.58

*S*_*ij*_	4	6	5	8	1	7	2	3

**Table 2:** Data for example problem.

*i*	*t*_*i*_	*d*_*i*_	*w*_*i*_	*r*_*i*_
1	2	10	2	1
2	3	15	5	3
3	5	20	6	4
4	4	10	3	2
5	6	10	1	2

**Table 3:** Working mechanism of neurodominance rule.

Solution	Total weighted tardiness	Result of NDR	Decision
**1**-**5**-4-2-3	134	0	Do not switch
1-**5**-**4**-2-6	134	1	Switch
1-4-**5**-**2**-3	119	1	Switch
1-4-2-**5**-**3**	85	1	Switch
1-4-2-3-5	46	—	—

**Table 4:** Computing total weighted tardiness (TWT).

*i*	*C*_*i*_ (complete time)	*d*_*i*_ (due date)	*T*_*i*_	*w*_*i*_	Weighted tardiness
1	3	10	0	2	0
2	15	15	0	5	0
3	24	20	4	6	24
4	9	10	0	3	0
5	32	10	22	1	22
Total weighted tardiness					46

*C*_*i*_: complete time of each job can be seen in Figure 2.

*T*_*i*_: tardiness, *T*_*i*_ = max⁡(0, (*C*_*i*_ − *d*_*i*_)).

TWT = *w*_*i*_ *∗T*_*i*_.

**Table 5:** Parameters of generated problems.

Elements	Distribution range
Processing time ranges	[1–10], [1–50], [1–100]
Weight ranges	[1–10], [1–50], [1–100]
Number of jobs	50, 70, 100, 120, 150, 200, 250, 300, 400
TF	0.1, 0.3, 0.5, 0.7, 0.9
RDD	0.1, 0.3, 0.5, 0.7, 0.9
*β*	0.0, 0.5, 1.0, 1.5

**Table 6:** Comparison of PSO and neurohybrid-PSO according to upper bound (TWT).

Number of jobs	Average of the best initial solutions	Upper bound for only using PSO		Upper bound for NHPSO		Effect of NDR
		After PSO	Improvement (%)	After hybrid systems	Improvement (%)	Improvement (%)
50	158256	141254	2.12	101698	2.38	0.05
70	152513	143574	2.58	99536	2.79	0.06
100	204415	165287	3.76	164118	3.98	0.06
120	242018	237521	4.02	235245	4.42	0.07
150	301764	297052	4.86	296027	5.12	0.09
200	402135	397233	5.64	394948	5.89	0.10
250	516287	511064	6.06	508420	6.45	0.12
300	609042	603245	6.69	598956	7.01	0.11
400	815371	809672	7.09	804896	7.82	0.12

**Table 7:** Comparison of GA and neurohybrid-PSO according to upper bound (TWT).

Number of jobs	Average of the best initial solutions	Upper bound for only using GA		Upper bound for NHPSO		Effect of NDR
		After GA	Improvement (%)	After hybrid systems	Improvement (%)	Improvement (%)
50	158256	144367	1.96	102048	2.46	0.06
70	152513	147085	2.02	100236	2.98	0.06
100	204415	179398	2.89	165284	4.25	0.07
120	242018	239125	3.58	236012	4.99	0.07
150	301764	299025	3.24	296983	5.83	0.08
200	402135	399889	4.22	396288	5.94	0.11
250	516287	513182	4.98	509121	6.98	0.12
300	609042	606032	5.33	599987	7.82	0.12
400	815371	812234	6.80	807348	7.96	0.12

**Table 8:** Comparison of SA and neurohybrid-PSO according to upper bound (TWT).

Number of jobs	Average of the best initial solutions	Upper bound for only using SA		Upper bound for NHPSO		Effect of NDR
		After SA	Improvement (%)	After hybrid systems	Improvement (%)	Improvement (%)
50	158256	144958	1.82	103257	2.59	0.06
70	152513	148025	1.96	106783	3.01	0.07
100	204415	176838	2.56	168021	4.14	0.06
120	242018	240528	2.89	237044	4.82	0.07
150	301764	298457	2.91	296875	5.99	0.08
200	402135	400569	3.58	395459	6.05	0.11
250	516287	514071	4.02	509149	6.99	0.11
300	609042	607822	4.88	599214	7.88	0.10
400	815371	813057	5.76	805648	7.98	0.12

**Table 9:** Comparison of linear lower bound results for PSO and NHPSO.

Number of jobs	Linear lower bound before PSO and NHPSO	Linear lower bound For only using PSO		Linear lower bound for NHPSO		Effect of NDR
		After PSO	Improvement (%)	After hybrid systems	Improvement (%)	Improvement (%)
50	850712	851034	0.621	851988	0.631	0.082
70	1211347	1212534	0.663	1212129	0.676	0.081
100	1829357	1831273	0.690	1831983	0.703	0.084
120	2139270	2140238	0.684	2141389	0.701	0.085
150	2653907	2654384	0.612	2654875	0.628	0.080
200	3109726	3110853	0.705	3111340	0.778	0.083
250	3467201	3468217	0.673	3469032	0.679	0.084
300	4103482	4107492	0.697	4108674	0.706	0.088
400	4976219	4977602	0.665	4978045	0.672	0.087

**Table 10 tab10:** Comparison of linear lower bound results for GA and NHPSO.

Number of jobs	Linear lower bound before GA and NHPSO	Linear lower bound For only using GA		Linear lower bound for NHPSO		Effect of NDR
		After GA	Improvement (%)	After hybrid systems	Improvement (%)	Improvement (%)
50	850712	851352	0.601	851884	0.636	0.081
70	1211347	1212048	0.628	1212856	0.682	0.082
100	1829357	1830639	0.630	1831238	0.705	0.083
120	2139270	2140112	0.672	2141022	0.722	0.087
150	2653907	2654026	0.528	2654135	0.631	0.078
200	3109726	3111492	0.637	3112027	0.782	0.072
250	3467201	3468939	0.593	3469251	0.682	0.083
300	4103482	4105832	0.603	4107336	0.710	0.086
400	4976219	4977012	0.598	4978879	0.683	0.088

**Table 11:** Comparison of linear lower bound results for SA and NHPSO.

Number of jobs	Linear lower bound before SA and NHPSO	Linear lower bound for only using SA		Linear lower bound for NHPSO		Effect of NDR
		After SA	Improvement (%)	After hybrid systems	Improvement (%)	Improvement (%)
50	850712	851262	0.588	851988	0.638	0.080
70	1211347	1212715	0.601	1212129	0.684	0.080
100	1829357	1830249	0.601	1831983	0.707	0.082
120	2139270	2140305	0.588	2141389	0.736	0.084
150	2653907	2654223	0.511	2654875	0.633	0.081
200	3109726	3110542	0.582	3110340	0.785	0.083
250	3467201	3468284	0.566	3469032	0.686	0.082
300	4103482	4104560	0.582	4108674	0.714	0.083
400	4976219	4977114	0.583	4978045	0.688	0.086

**Table 12:** Results of linear lower bound for 810000 examples (PSO).

Number of jobs	Better	Equal	Worst	Total	*t*-test
50	4237	85437	326	90000	5.88
70	9846	79322	832	90000	5.34
100	7645	78765	3590	90000	5.22
120	6953	81751	1296	90000	6.37
150	7021	79730	3249	90000	5.69
200	7112	80015	2873	90000	7.48
250	7745	81221	1034	90000	5.32
300	6903	78881	4216	90000	6.01
400	6766	79390	3844	90000	6.22

**Table 13 tab13:** Results of linear lower bound for 810000 examples (GA).

Number of jobs	Better	Equal	Worst	Total	*t*-test
50	4312	85344	344	90000	6.01
70	9923	79165	912	90000	5.77
100	7836	78542	3622	90000	5.36
120	6986	81689	1325	90000	4.82
150	7231	79457	3312	90000	5.23
200	7236	79801	2963	90000	6.86
250	7822	81066	1112	90000	4.87
300	6996	78715	4298	90000	5.04
400	6793	79282	3925	90000	4.93

**Table 14:** Results of linear lower bound for 810000 examples (SA).

Number of jobs	Better	Equal	Worst	Total	*t*-test
50	4344	85305	351	90000	5.11
70	9901	79274	852	90000	6.27
100	7873	78483	3644	90000	4.36
120	7001	81666	1333	90000	7.02
150	7288	79330	3382	90000	6.33
200	7301	79712	2987	90000	4.26
250	7944	80931	1125	90000	5.68
300	7012	78686	4302	90000	4.48
400	6832	79212	3956	90000	5.01
